# Intercomparison of Local Warming Trends of Shanghai and Hong Kong Based on 120-Year Temperature Observational Data

**DOI:** 10.3390/ijerph19116494

**Published:** 2022-05-26

**Authors:** Yawei Yang, Lei Li, Pak-Wai Chan, Qianjin Zhou, Bosi Sheng

**Affiliations:** 1 Southern Marine Science and Engineering Guangdong Laboratory (Zhuhai), School of Atmospheric Sciences, Sun Yat-sen University, Zhuhai 519082, China; yangywsh@163.com (Y.Y.); zhouqj8@mail2.sysu.edu.cn (Q.Z.); shengbs@mail2.sysu.edu.cn (B.S.); 2 Shanghai Climate Center, Shanghai 200030, China; 3 Guangdong Provincial Observation and Research Station for Climate Environment and Air Quality Change in the Pearl River Estuary, Zhuhai 519082, China; 4 Ministry of Education Key Laboratory, Atmosphere-Ocean System, Sun Yat-sen University, Zhuhai 519082, China; 5 Hong Kong Observatory, Hong Kong 999077, China; pwchan@hko.gov.hk

**Keywords:** urban climate, local climate, urbanisation, warming, Shanghai, Hong Kong

## Abstract

Using surface air temperature observations from 1901 to 2020, this study compared the warming trends of Shanghai and Hong Kong over a period of 120 years. The statistical results reveal the following: (1) The average temperatures of the two cities underwent fluctuating increases during the past 120 years, with linear warming rates of 0.23 °C/decade in Shanghai and 0.13 °C/decade in Hong Kong. (2) The fluctuation ranges of maximum temperature in the two cities were considerably higher than those of mean temperature. Moreover, in both cities, the annual mean maximum temperature decreased during a phase of more than a decade. The fluctuation ranges of minimum temperature were smaller, whereas the linear increases were higher than those for the mean temperature. (3) The diurnal temperature ranges (DTRs) of the two cities decreased; a certain phase of the decreases in DTR in the two cities was caused by decreases in the maximum temperature. (4) At a certain stage of urban development, owing to the shading effect of new high-rise buildings, the solar shortwave radiation reaching the Earth’s surface decreased, and anthropogenic heat generated by the energy consumption of buildings and urban human activities at that time was not sufficient to make up for the reduced shortwave radiation. This result may have led to the declines in the maximum temperature experienced by both cities. (5) Currently, the number of hot days and extremely hot days in the two cities has increased significantly compared with that a century ago, indicating that climate warming has an adverse impact on human settlements.

## 1. Introduction

Global warming and urbanisation are two important factors that affect the local climates of urban areas. According to the Intergovernmental Panel on Climate Change (IPCC), the pace of global warming has continued unabated since the Industrial Revolution [1], impacting the background climates of regions where most cities on Earth are located. Moreover, urbanisation, characterised by population growth, land expansion, and land surface change [2,3], has a significant impact on the local climate [4]. A deeper understanding of the effects of urbanisation on local climate and its related mechanisms is required to better plan and design climate-friendly cities [5].

Urbanisation has significantly changed the physical properties of the underlying surface of cities, including specific heat capacity. These changes, together with the anthropogenic heat emitted by human activities in urban areas, have significantly changed the energy balance between the atmosphere and the Earth’s surface, thus significantly affecting the local climate [6,7]. In the early 19th century, British scientist Luke Howard first proposed the urban heat island phenomenon in his famous book *The Climate of London*, which means that the average temperature in urban areas was higher than that in the rural areas. Some studies have found that in sunny and calm wind conditions, cities can be up to 12 degrees warmer than suburban areas because of the urban heat island effects [8], albeit in extreme cases. In tropical or subtropical cities, the increasingly hot climate has an adverse impact on the health of residents. With local climates, the rate of warming is closely related to the rate of urbanisation. China has been one of the most rapidly urbanising countries in the world for the past 40 years [9,10]. The impact of this urbanisation on the local climates of cities has attracted extensive attention and spawned a large number of studies [11,12,13]; some have pointed out that in cities with very rapid urbanisation, such as Shenzhen, urbanisation’s contribution to warming is greater than 80% [14].

Shanghai and Hong Kong are two major Chinese cities. Both cities have experienced rapid development in the past century, have become highly modernised, and are currently important East Asian financial centres and seaports. Although the effects of urbanisation on these two cities are evident, it is useful to study their local climate change characteristics to more fully understand the climate effects of urbanisation. The two cities are located at different latitudes, with Shanghai at around 31°12′ and Hong Kong at around 22°18′. Due to the difference of geographical location, there is a certain gap in the statistical values of climate elements between the two cities. According to the statistical data of the last 30 years (1991–2020), the average annual temperature in Shanghai is 17.5 °C and the average annual rainfall is 1336.6 mm, while the average annual temperature in Hong Kong is 23.5 °C, and the average annual rainfall is 2431.2 mm. However, the two cities still have many similarities. For example, they are both located in the East Asian subtropical monsoon climate zone, they are both coastal cities, and they both have extremely high economic and population densities. These similarities make their climate change trends somewhat comparable. More importantly, these two cities were two of the first cities in East Asia to conduct surface meteorological observations, with available data spanning more than a century, allowing us to study the climate effects of their urbanisation.

Shanghai and Hong Kong are also quite different, especially in terms of their respective development histories. Although Shanghai has long been a major city in east China, its development history has been tortuous. During the 1930s and 1940s, Shanghai was plunged into the chaos of war. After the founding of the People’s Republic of China in 1949, its development environment stabilised, and it gradually became an important industrial city. In the 1980s, following the implementation of the policy of reform and opening up in China, Shanghai experienced a period of rapid development, marked by high levels of economic development and urbanisation. Hong Kong, on the other hand, was a British colony for many years, and, except for a period of Japanese occupation during World War II, its development was generally stable. For a long time, as the Hong Kong government attached great importance to environmental protection, it placed strict controls on expanding urban built-up areas. As a result, urban density continuously increased in Hong Kong, especially after the 1970s with the proliferation of high-rise buildings to accommodate the growing population.

Previous studies have looked at the urban climates of the two cities separately. Chen et al. [15] analysed the impact of urbanisation on the climate of the Pearl River Delta using meteorological observation data from Hong Kong. Chan et al. [16] analysed temperature changes in Hong Kong between 1971 and 2010, with the focus on seasonal changes. Li et al. [14] analysed changes in temperature, humidity, and rainfall in Hong Kong from 1968 to 2013, using data from Hong Kong to contrast the impact of Shenzhen’s rapid development on the local climate. Chow [17] analysed the urban climate characteristics of Shanghai, focusing on the urban heat island effect in Shanghai. Similarly, Zhang et al. [18] analysed the spatial and temporal characteristics of the urban heat island in Shanghai. These studies significantly improved the scientific understanding of the climate characteristics of the two cities and contributed to the field of urban climate as a whole. Few studies, however, have conducted a detailed analysis of the observation data of the two cities over a study period exceeding a hundred years; therefore, the scientific value of the climate effect of urbanisation that the data can reveal has not been fully uncovered. No study has compared long-term observational data from the two major East Asian cities of Shanghai and Hong Kong; such work can advance scientific understanding of the climate effects of urbanisation in the region.

To this end, this study used surface temperature and precipitation observation data for Shanghai and Hong Kong to conduct statistical analysis of the climate change trends of the two cities over the past 120 years in an attempt to improve understanding of the role of urbanisation in these changes.

## 2. Data and Method

**Figure 1:**
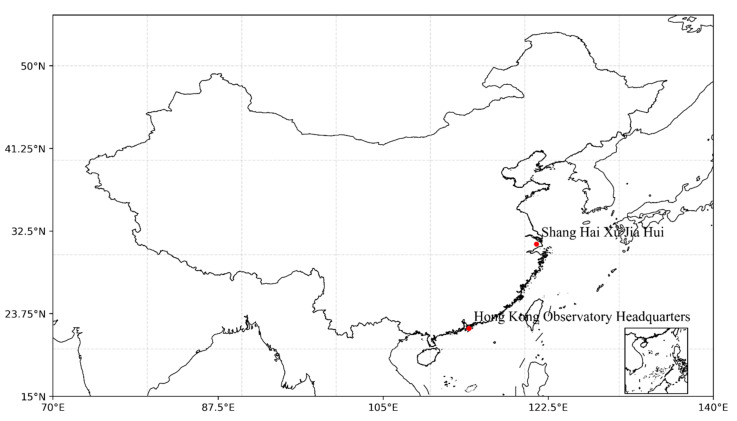
The locations of the two observatories.

The data used in this study are from the Xujiahui Observatory in Shanghai (SXO) and the Hong Kong Observatory (HKO) headquarters in the city’s Tsimshatsui district and consist of three types of daily temperature observations: daily average temperature, daily minimum temperature, and daily maximum temperature. The data span from 1 January 1901 to 31 December 2020. The locations of the two sites are shown in Figure 1. The location of HKO headquarters, on a peninsula surrounded by the sea, has not changed during this period. Meanwhile, the SXO had two significant migrations during the study period, which occurred on 1 January 1956 and 1 July 1999, respectively. The distance of the migration was no more than 3 km, and the latter migration was actually moved back to the original site before the relocation in 1956. Both of the SXO locations are around 20 km away from the coastline. Theoretically, the first migration may have a more obvious impact on the temperature observation because it moved from the built-up area to a relatively open suburb nearly 3 km away, so there may be a sudden change in the nature of the observation environment. When the station was relocated for the second time, the original suburbs had already been transformed into urban areas during the urbanization process, and the new relocation was to move back to the old site of similar built-up area.

Notably, owing to the disruption during World War II, data for Hong Kong was not collected for seven years, from 1940 to 1946. To cross-check the data, this study used the Land Surface Air Temperature (LSAT) dataset, which is a gridded temperature dataset developed by Cheng et al. [19], with data spanning over a period from 1901 to 2018, resolution of 0.5° × 0.5°, and temporal resolution of one month. This study also used observational environment metadata from the HKO headquarters [20] as a crucial factor for understanding Hong Kong’s changing air temperature.

Based on daily observation data, the following annual statistical data were calculated: the annual mean temperature, annual mean diurnal temperature ranges (DTRs), annual mean daily maximum temperature, annual mean daily minimum temperature, annual mean daily temperature, number of hot days per year above 33 °C, and number of extremely hot days per year above 35 °C. Daily DTR is calculated by subtracting the daily minimum temperature from the daily maximum temperature. Based on the calculated annual statistics, we obtained statistical data for different 30-year periods. We chose 30 years since the statistical period the World Meteorological Organisation uses the average of climatological elements for a uniform 30-year period as the standard climatological normal of a region. In this study, we calculated differences for the various elements in the last 30 years (1991–2020) and the first 30 years (1901–1930) to help understand changes in the climates of the two cities compared to a hundred years ago.

It should be mentioned that although SXO has been relocated twice during the period of study, the data homogenization has not been conducted. The current study intends to discuss the characteristics of local climate change based on the original raw data. However, when trying to analyse the causes leading to various changes of the long-time series of temperature data, the station relocation will be considered as one important factor.

A linear least squares regression (LLSR) method is used to analyse the warming trend of average temperature. The basic equation for LLSR method is:

(1) Y=a+bX

where *X* is predictor (year in the current study) and *Y* is predictand (temperature). The coefficients *a* and *b* are calculated as follows:

(2) $$a=\bar{y}-b\bar{x}$$

(3) $$b=\frac{\sum_{i=1}^{n}x_{i}y_{i}-n\bar{x}\bar{y}}{\sum_{i=1}^{n}x_{i}^{2}-{\bar{x}}^{2}}$$

where $\bar{x}$ and $\bar{y}$ are average values of the two data series; *n* is the size of the sample; *x_i_* and *y_i_* are the samples of the predictor and predictand. The value of *b* also indicates the trend of *y*. Finally, the correlation coefficient *R* of the linear regression is computed to evaluate the confidence level from the obtained trend by using LLSR method. The equation for calculating *R* is as follow:

(4) $$R=\frac{\sum_{i=1}^{n}\left(x_{i}-\bar{x}\right)\left(y_{i}-\bar{y}\right)}{\sqrt{\left(\sum_{i=1}^{n}{\left(x_{i}-\bar{x}\right)}^{2}\right)\left(\sum_{i=1}^{n}{\left(y_{i}-\bar{y}\right)}^{2}\right)}}$$

## 3. Results and Analysis

### 3.1. Average Air Temperature

**Figure 2:**
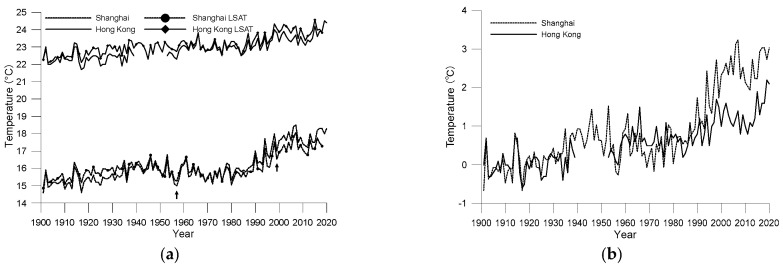
Annual average air temperature from 1901 to 2020 for the Shanghai and Hong Kong observatories ((**a**) original observations; (**b**) anomalies to the average values during 1901–1930).

Figure 2a shows the changes in annual mean temperature in Shanghai and Hong Kong between 1901 and 2020. It should be noted that for the curves of Shanghai, the time points of the two relocations are marked with arrows. It can be seen from Figure 2 that although both cities are in the subtropical monsoon climate zone, owing to their different latitudes, the difference in annual average temperature between the cities is approximately 7 °C. Additionally, similarities exist in the temperature change curves of the two cities, as both have upward fluctuating trends over the past 120 years. The slope of the linear trend of annual mean temperature is 0.23 °C/decade in Shanghai and 0.13 °C/decade in Hong Kong, significant at the 0.01 confidence level. According to an IPCC assessment, the average global surface temperature has risen by approximately 1 °C since 1850–1900 [1]; therefore, both cities warmed faster than global warming. The warming effect of urbanisation may have played an important role.

Figure 2a shows that there is a gap between the two time series of the two cities, which is the systematic difference of the two time series and is mainly determined by the difference of background climate induced by the difference of total solar radiation received at different latitudes. By subtracting the climate values from the annual average temperature, the anomalies of the temperature relative to the climate values can be obtained. When comparing the anomalies, the influence of the climate value has been eliminated, thus removing the systematic errors. Figure 2b shows anomalies to the annual mean temperatures of the two cities from 1901 to 1930; it shows the warming histories of the two cities more clearly because systematic differences caused by latitude are removed. Figure 2b shows that before the 1980s, the two cities had roughly the same pace of warming, differing after the 1980s. The average mean temperature in Shanghai increased rapidly after 1980, while the trend in Hong Kong was less convoluted, with a gradual but more sustained upward trend. In a recent study, Hou et al. [21] pointed out that the observed temperature trend in Metropolis of East China is around 0.90 °C/decade since 1981. Meanwhile, as estimated by Chen et al. [15], the temperature trend in PRD area since 1984 is around 0.82 °C/decade, which is less than that of East China. The results of the current study are accordant to the previous studies. The reason leading to the difference in temperature trend between the two cities may be related to both background regional climate variation and local urbanization characteristics.

Figure 2a shows the time series of annual mean temperatures at the grid points of Shanghai and Hong Kong based on LSAT data. It can be seen from the figure that although the temporal (monthly) and spatial (0.5° × 0.5°) resolutions of the LSAT data are not very refined, the trends are consistent with those in the surface observation data of the two cities, with similarities in both the long-term trends and interannual fluctuations. This result sufficiently demonstrates that the accuracy of the data from the two sources is relatively reliable and they can be used to verify each other. Especially for Shanghai, although no homogenization has been conducted to the original data, its change trend is still highly consistent with LSAT data. Understandably, gridded data will still contain errors from surface observations because gridded data represent values in a 0.5° × 0.5° area, while surface observations are observations made at a single point.

For Hong Kong, the values from LSAT are always higher than the surface observation data; this finding indicates that the surface temperature in Hong Kong maintains a rhythm similar to the regional temperature changes. The temperature rise rates calculated from the data from the two sources are almost identical. However, the situation in Shanghai is different. The air temperature values from the surface observation data in Shanghai were lower than those from the LSAT data before the 1950s but higher than those from the LSAT data after the 1980s. Regarding the linear trends, the linear temperature rise rate of Shanghai according to LSAT data is 0.13 °C/decade, lower than that of the surface observation station. This difference between Hong Kong and Shanghai indicates differences in the impact of urbanisation on single-point observation data in the two cities.

In view of the lack of observation data in surrounding areas, it is impossible to homogenize the observation data of Shanghai directly in this study. However, the LSAT dataset itself is a very strictly homogenized one. Therefore, without the support of other reliable data sources, it is still meaningful to use LSAT as a reference to verify the reliability of the data of Shanghai used in this study. In order to verify the reliability of the data of Shanghai, four groups of data are used. Group A is the original ground observation data of Shanghai, group B is the LSAT data of the grid cell where the Xujiahui Observatory in Shanghai is located, group C is the interpolated data from nine surrounding grid cells to position 1 (valid for 1901–1956 and 1999–2020), and group D is the interpolated data from nine surrounding grid cells to position 2 (valid for 1957–1998). The comparison between the values of the data of B, C, and D obtained from LSAT are very close to each other, and the variation trend of the original observation data is also similar to them. The average value of relative error and correlation coefficient between different groups of data are calculated, and the results are shown in Table 1. It can be seen from Table 1 that the relative errors between the observation data (group A) and the grid data of different groups (groups B, C, and D) are very small; the maximum value is only 2.02%. The correlation coefficients between observation data and gridded data all exceed 0.95, which can pass the significance test of 0.01 confidence. The data of group C and D are for the two locations before and after the station relocation, respectively, and the relative error between them is 0.11%, and the correlation coefficient is as high as 0.99997, which shows that the impact of site relocation on the observation data is insignificant.

### 3.2. Comparison of Average Temperature between Urban and Rural Areas

**Figure 3:**
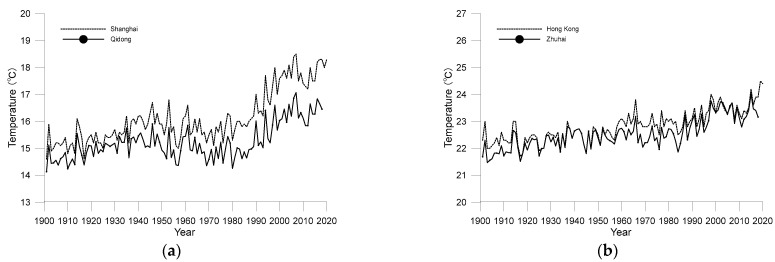
Comparison of annual average air temperature between the two target cities and their adjacent rural areas from 1901 to 2020 ((**a**) Shanghai and Qidong; (**b**) Hong Kong and Zhuhai).

In order to analyse the impact of urbanization on the temperature of the two cities, it is necessary to compare the temperature changes between urban and rural areas. However, as mentioned earlier, these two cities are the first cities to carry out observation in this region, so it is impossible to find rural station observation data matching their time scale. Fortunately, the LSAT dataset provides data resources with a long enough time span and covering the entire Chinese Mainland. The data of two grid points are extracted from the LSAT dataset. The grid point located in Qidong City is selected for comparison with Shanghai, which is about 90 km north to SXS. The grid point selected as background of Hong Kong is located in Zhuhai, which is about 70 km west to HKO. The two points are not only close to the target cities, but also have almost the same background climate characteristics as their target cities. The start-up of development of the two background areas is later than that of Shanghai and Hong Kong, which is helpful to analyse the impact of urbanization on the temperature trend of the target cities. The comparison of the average annual temperature between the two target cities and their adjacent rural areas is shown in Figure 3.

Figure 3a shows that the urban heat island effect in Shanghai was obvious at the beginning of the 20th century, and its average annual temperature is steadily higher than that in adjacent rural areas. The trend of temperature change in the Qidong area calculated by LLSR method is about 0.12 °C/decade, which is less than 0.23 °C/decade in Shanghai. However, Figure 3b shows that the urban heat island effect in Hong Kong was more obvious in the 1910s and 1950–1980, but not in other periods. Especially after 1980, with the implement of China’s reform and opening-up policy, the urbanization process of the whole Pearl River Delta accelerated, Zhuhai, as the background area, also developed rapidly, and the average temperature soon caught up with Hong Kong. This characteristic is similar to what has been found in the comparative study of temperature increase between Hong Kong and Shenzhen [14]. Throughout 1901–2018, the temperature change trend of Zhuhai is about 0.13 °C/decade, which is roughly equivalent to that of Hong Kong and Qidong, the background city of Shanghai. It should be noted that the temperature change trends of the two background stations are significant at a confidence level of 0.01. The whole Figure 3 shows that although the two cities have many similarities, since 1901, the impact of urbanization on the warming of Shanghai is greater than that of Hong Kong overall.

### 3.3. DTR

DTR is an important parameter that reflects the influence of urbanisation on air temperature. Some studies have pointed out that as urbanisation progresses, DTR usually decreases, mainly due to a rapid rise in the daily minimum temperature [14,15,22]. It is generally believed that a decrease in DTR has an adverse impact on the living environment of cities in subtropical regions. This situation is particularly observed in summer when a lower DTR makes cooling down at night difficult following hot weather during the day, thus reducing people’s comfort level. Another study shows that DTR is closely related to non-accidental mortality. A one-degree increase in DTR at lag 0–4 days was associated with a 0.47% (95% confidence) increase in non-accidental mortality [23].

**Figure 4:**
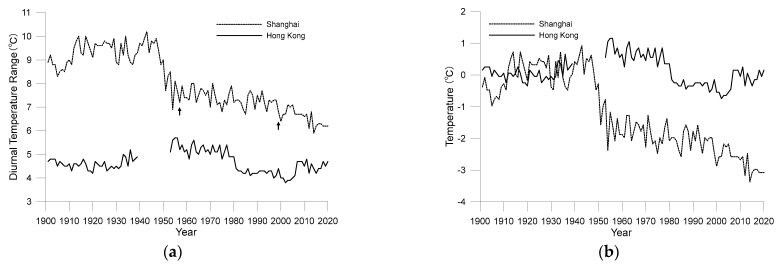
Annual average diurnal temperature range from 1901 to 2020 for the Shanghai and Hong Kong observatories ((**a**) original observations; (**b**) anomalies to the average values during 1901–1930).

Figure 4a shows the DTR of Shanghai and Hong Kong from 1901 to 2020. As shown in the figure, Hong Kong’s DTR was lower than that of Shanghai owing to the difference in geographical locations of the two observatories. Tsimshatsui in Hong Kong is located on a peninsula surrounded by sea on three sides, at a straight-line distance of only 2 km from the coast. The temperature observed here is significantly affected by the surrounding ocean. However, as the distance between Xujiahui in Shanghai and the coast is more than 20 km, the observed temperature is less affected by the ocean.

Figure 4b shows the anomalies in the annual mean DTR relative to the mean DTR of 1901–1930. Importantly, over the past 120 years, the DTR of both cities decreased somewhat, but their respective fluctuations were different. Shanghai’s DTR dropped sharply from the late 1940s, from 9.4 °C in 1948 to 6.9 °C in 1954. A fluctuating downward trend continued, but the rate of decline was considerably low, dropping to around 6.2 °C by 2020. The decline in Hong Kong’s DTR began in the 1950s, but the rate of decline did not accelerate until the mid-1970s. In the 2000s, Hong Kong’s DTR increased slightly.

The DTR changes in Shanghai and Hong Kong indicate that decreased DTR due to urbanisation is a common phenomenon; however, specific changes, such as those that may be related to the geographical location of observation sites and those in the surrounding environment of observation sites, occurring during urbanisation can differ.

### 3.4. Maximum and Minimum Air Temperatures

**Figure 5:**
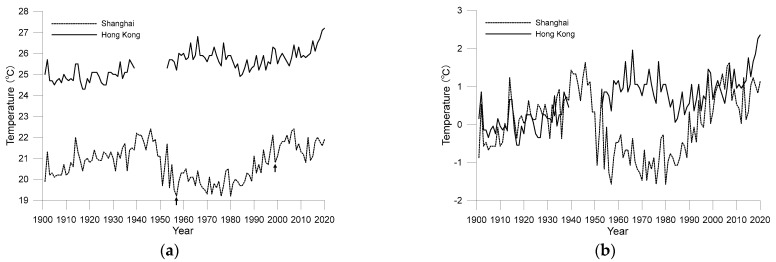
Annual average maximum temperature from 1901 to 2020 for the Shanghai and Hong Kong observatories ((**a**) original observations; (**b**) anomalies to the average values during 1901–1930).

**Figure 6:**
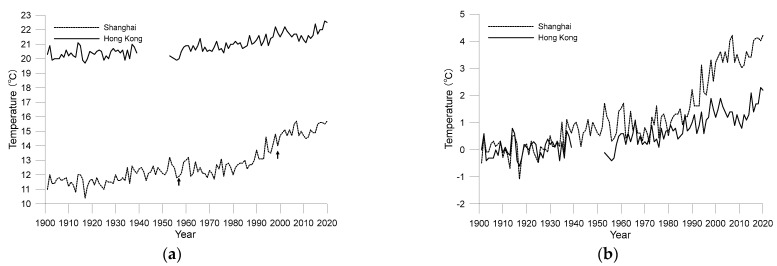
Annual average minimum temperature range from 1901 to 2020 for the Shanghai and Hong Kong observatories ((**a**) original observations; (**b**) anomalies to the average values during 1901–1930).

Figure 5 and Figure 6 show changes in annual mean maximum and minimum temperatures for Shanghai and Hong Kong from 1901 to 2020 and their deviations from the corresponding mean values for 1901–1930. It can be seen from the two figures that fluctuations in annual mean maximum temperature in the two cities were more complex than those in the minimum temperature.

In Shanghai, the maximum temperature rose slowly between 1901 and the mid-1940s, but there was a sharp decrease beginning in the late 1940s, rising again from 1980 onwards. The current annual mean maximum temperature in Shanghai is approximately 21.5 °C, which is about 2 °C higher than the low points in the past 120 years. The trend of minimum temperature in Shanghai is more straightforward, with an overall continuous upward trend. There was a slight downward trend from the mid-1940s, but it was less pronounced. After 1980, the rise in minimum temperature in Shanghai accelerated significantly, exceeding the rise in the maximum temperature.

Similar to Shanghai, the trend of maximum temperature in Hong Kong also rose, fell, and rose again, but the falling period differed from that of Shanghai, occurring in the mid-1960s to the early 1980s. In addition, the decline in the maximum temperature in Hong Kong was not as pronounced as in Shanghai, causing it to be noticed and mentioned less in the past. The increase in Hong Kong’s minimum temperature was relatively steady, with no sharp rise after 1980, as was observed in Shanghai.

As noted earlier, the overall decrease in DTR in the cities was largely attributable to a rapid rise in daily minimum temperatures. However, comparing Figure 4, Figure 5 and Figure 6, an interesting conclusion can be made that the decline in urban DTR cannot be simply attributed to a rapid rise in minimum temperature. For example, in Shanghai, the decrease in DTR from the mid-1940s to the end of the 1970s was mainly caused by a decrease in the maximum temperature. The increase in Shanghai’s minimum temperature was not significant during that period. After 1980, the fall in Shanghai’s DTR occurred due to the rise in minimum temperature being faster than the rise in the maximum temperature. Similarly, the drop in DTR in Hong Kong between the early 1960s and early 1980s was also caused by a drop in the maximum temperature.

**Figure 7:**
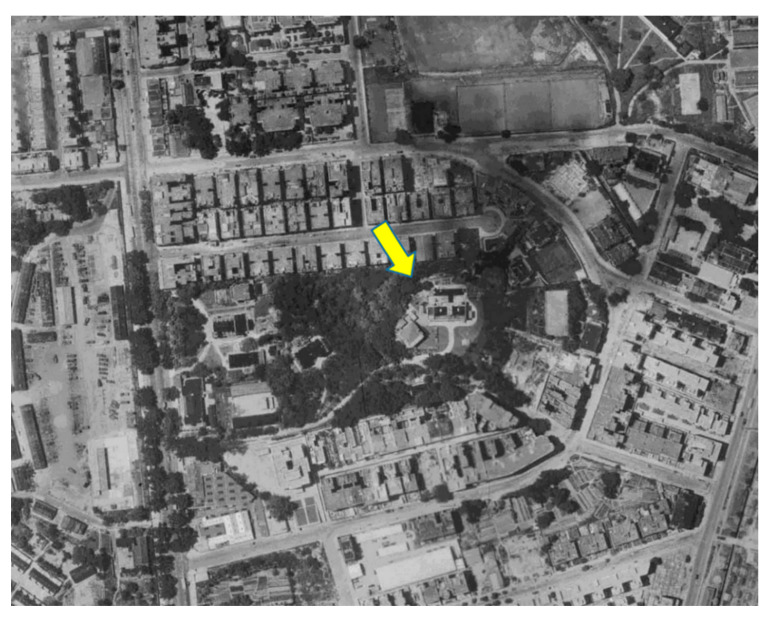
Buildings surrounding the HKO headquarters in May 1949. The yellow arrow indicates the location of the Hong Kong Observatory, the same below.

**Figure 8:**
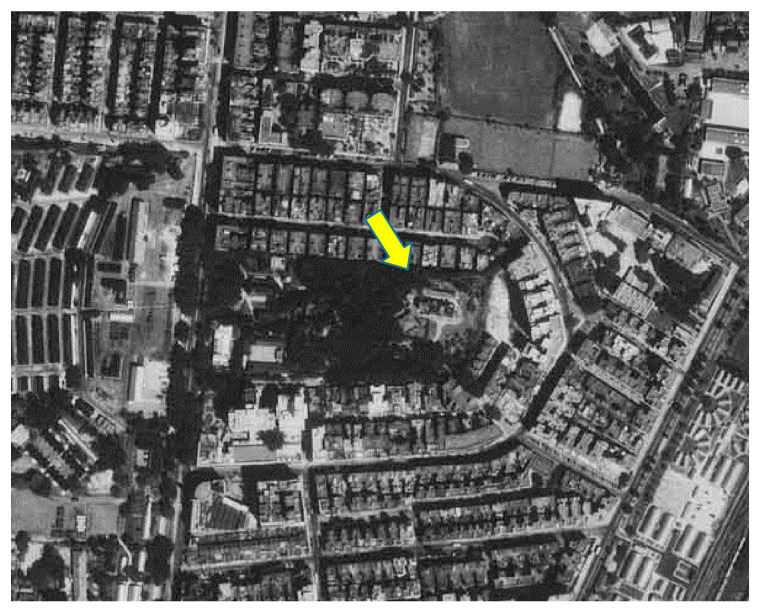
Buildings surrounding the HKO headquarters in October 1959.

**Figure 9:**
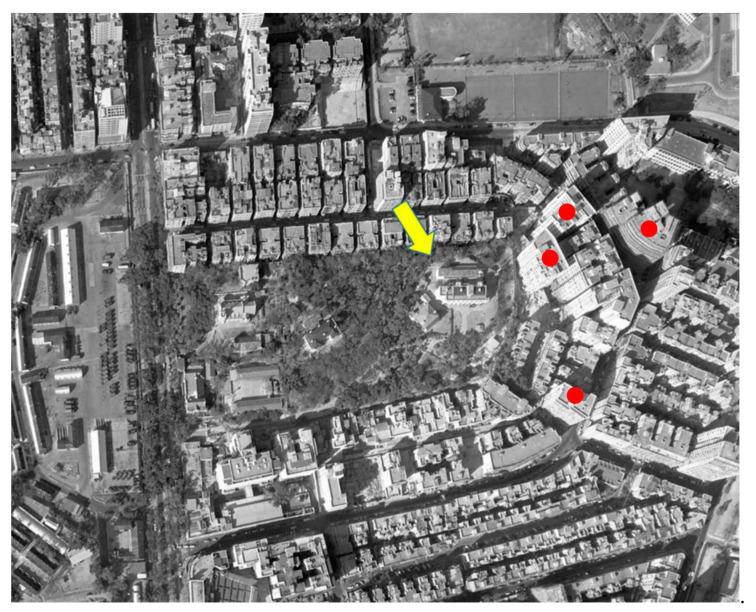
Buildings surrounding the HKO headquarters in January 1963, red dots indicate new tall buildings appeared after 1959.

An important question is: what caused the significant drops in the maximum temperature in the course of urbanisation? To better understand the underlying reasons, this study analysed the problem from the perspective of the observational environments of the sites. A report by the HKO headquarters on long-term changes in the metadata of the observational environment provided the basic information needed to answer this question. Figure 7, Figure 8 and Figure 9 depict aerial photographs showing the buildings around the HKO headquarters in different periods, obtained from the report by Lee et al. [20]. Although it is difficult to determine the exact heights of the buildings from these images, it is possible to qualitatively evaluate changes in the observational environment. Figure 7 shows the site in 1949. Although there were already some buildings around the HKO headquarters at that time, the buildings were generally relatively low-rise. Figure 8 shows the site in 1959. It can be seen that there are already many high-rise buildings around the HKO headquarters, especially on the east and south sides. By 1963, the number of high-rise buildings surrounding the observatory increased. The red dots in Figure 9 indicate the new high-rise buildings compared to 1959. The new high-rise building closest to the HKO headquarters significantly reduced the distance between the observation point and surrounding high-rise buildings.

**Figure 10:**
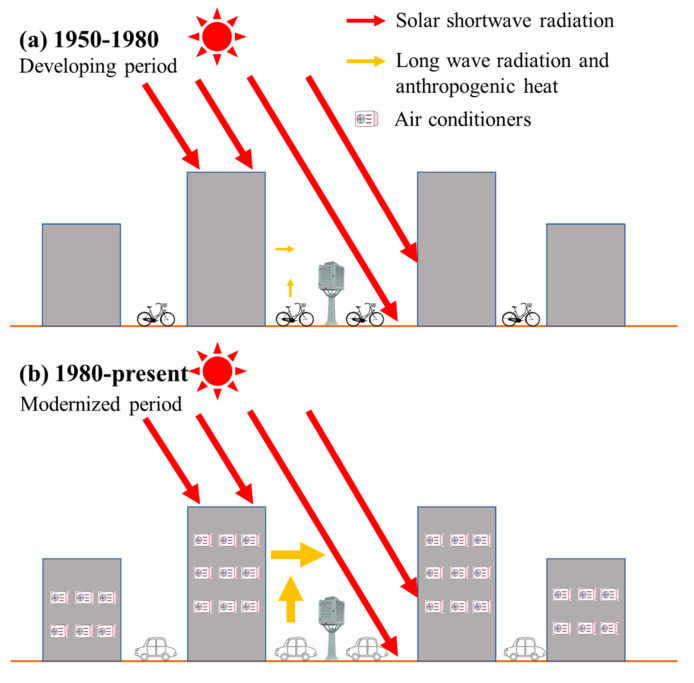
Physical mechanism of the evolution of the maximum temperature in Hong Kong ((**a**) 1950–1980, with little anthropogenic heat; (**b**) 1980–present, with strong anthropogenic heat. The thickness of arrows indicates the strength of radiation energy).

The increase in the number of high-rise buildings around the observation point had various impacts on the temperatures observed at the HKO headquarters. At night, the main effect was the increase in minimum temperature, mainly due to interreflections between buildings, which can trap longwave radiation energy inside the urban canopy, heating the air within the canopy. In addition, the anthropogenic heat released by buildings, even if not intense, also heats the air. During the day, the situation is more complicated. High-rise buildings will block solar shortwave radiation, significantly reducing the energy that reaches the surface. In addition, longwave radiation reflected between buildings and anthropogenic heat are still present, which will also heat the atmosphere within the canopy. Given that interdecadal variation in longwave radiation from buildings is not too severe, the impact of buildings on the maximum diurnal temperature depends somewhat on the ratio of reduced shortwave radiation to increased anthropogenic heat. Taking the above considerations into account, Figure 10 presents a possible mechanism explaining the brief drop in mean maximum temperature in Hong Kong after the 1960s and the sustained increase after the 1980s. As shown in Figure 10a, from the late 1950s to the early 1960s, the large number of high-rise buildings shielded the surface from a significant proportion of solar shortwave radiation, leading to a gradual decrease in the mean maximum temperature during this period. From the 1980s onwards, as the number of motor vehicles and building energy consumption in the city increased, the resulting increase in anthropogenic heat gradually offset the decrease in shortwave radiation. This effect was compounded by continuing global warming, causing the mean maximum temperature in Hong Kong to rise again.

Finally, it should also be pointed out that the change trend of the maximum temperature in Shanghai is unusual, especially the decline during the mid-1940s to the 1950s, which is more drastic than the decline trend in Hong Kong. Although this paper suggests that the decline in this period may be related to the shielding of short-wave radiation brought by urbanization, it may also be related to other factors, including the replacement of sensors and the shielding measures on the temperature observation point, which may also affect the observation results of the maximum temperature. However, due to the lack of metadata in SXO, it is difficult to trace its truth.

It is difficult to explain similar changes in Shanghai’s maximum temperature accurately owing to a lack of long-term metadata on the observational environment. Nevertheless, based on the situation in Hong Kong, we can infer that the same mechanism existed in Shanghai. In particular, the oscillating decline trend of the maximum temperature during 1945–1956 cannot be explained by the station relocation in 1956. A more likely explanation is that the surrounding areas of Xujiahui, as the city centre, began to be occupied by new high-rise buildings during this period, reducing the short-wave radiation over the SXO site, resulting in the reduction of the maximum temperature. It was the deterioration of the observation environment that led to the relocation of SXO in 1956. After moving into the new site, due to the distance from the built-up area, the impact of urban anthropogenic heat was significantly reduced, and the low level of the average annual maximum temperature could be maintained. The new site was gradually surrounded by the expanding built-up area during the period of 1956 to 1980. By 1980, this area was basically located in the middle of the urban built-up area again. At the same time, with the implementation of the reform and opening-up policy, Shanghai’s urban energy consumption increased rapidly to support the development of the city and compensated the possible loss of short-wave radiation caused by the shielding of tall buildings, and consequently the maximum temperature rebounded rapidly. The second relocation in 1999 did not change the underlying surface properties of the observation station (maintaining the urban built-up area), and the distance from the original site was less than 3 km, so the rising trend of the maximum temperature was maintained.

### 3.5. Comparison of Temperature Variation Trends between Winter and Summer

**Figure 11:**
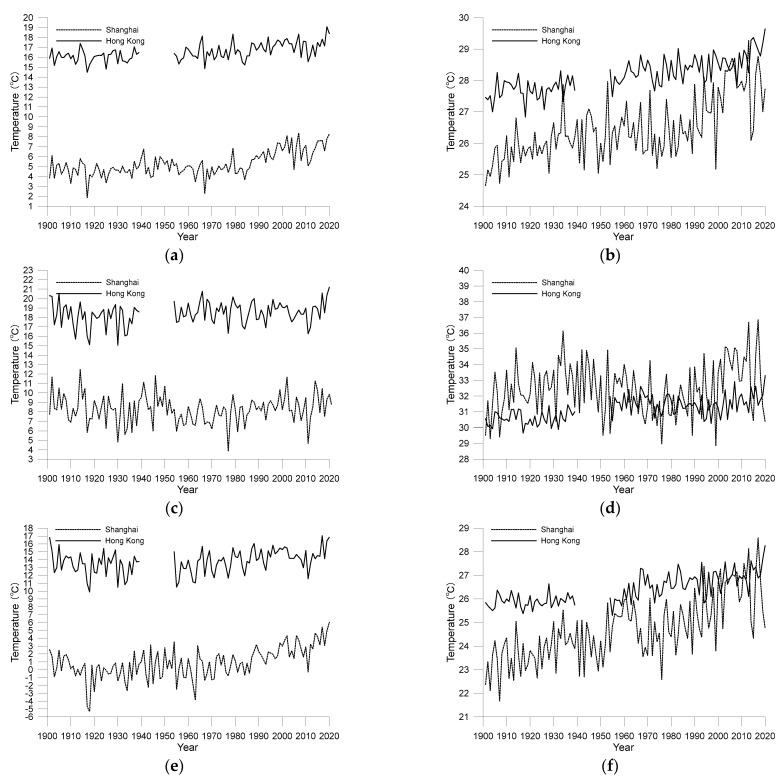
Temperature variation trends in different seasons from 1901 to 2020 for the Shanghai and Hong Kong observatories ((**a**) the average temperature in winter; (**b**) the average temperature in summer; (**c**) the maximum temperature in January; (**d**) the maximum temperature in July; (**e**) the minimum temperature in January; (**f**) the minimum temperature in July).

In order to better analyse the impact of urbanization and environmental changes on temperature, Figure 11 compares the average temperature in summer (December, January, and February, DJF) and winter (June, July, and August, JJA), as well as the maximum temperature and minimum temperature in January and July.

Figure 11a,b show the variation of average temperature in winter and summer from 1901 to 2020, respectively. It can be found that the difference of latitude has a very significant impact on the seasonal average temperature of the two cities. The temperature difference between the two cities in winter is much greater than that in summer. In winter, because the sun shines directly on the southern hemisphere, the difference of the received solar radiation between the two cities in the northern hemisphere is more obvious. In winter, Shanghai receives much less radiation because it is located in the area with higher latitude and lower solar inclination, resulting in a much lower average temperature. In summer, the sun shines directly on the northern hemisphere, and the difference of solar radiation between the two cities is not as obvious as that in winter, so the temperature difference between them is also smaller. Figure 11a,b illustrate the importance of changes in solar radiation intensity to changes in temperature. Another possible reason leading to the difference of the seasonal average temperature is the geographical locations of the two observatories. SXO is farther away from the seashore, and the ocean’s regulating effect on the observed temperature at SXO is weaker, so the temperature difference between summer and winter is greater. HKO is located on the peninsula surrounded by sea, and the ocean has a more significant regulating effect on the temperature at HKO, so the temperature difference between summer and winter is relatively small.

Figure 11c,d show the maximum temperatures in January and July, respectively. It can be seen from the figure that compared with the average temperature, the difference between the maximum temperatures in the two cities is more obvious in different seasons. Especially in July, the maximum temperature in Shanghai is even higher than that in Hong Kong, which is at lower latitude. Figure 11c,d show that although the fluctuation of the maximum temperature in January is large, it is not easy to observe an obvious linear change trend. Meanwhile, the trend of the maximum temperature in July is relatively more obvious, which is more consistent with the trend of the annual average maximum temperature. Especially for Shanghai, the downward trend of the maximum temperature during 1940s and 1950s is the focus of attention. Figure 11d shows that during the period, the decline of the maximum temperature in July exceeded 5 °C, which is greater than that of the annual maximum temperature in the same period (3 °C). This comparison supports the previous speculation that the short-wave radiation was possible to be significantly shielded during this period, resulting in a significant decrease in the maximum temperature.

Figure 11e,f are the minimum temperatures in January and July, respectively. It can be seen from the figure that the trend of the minimum temperatures is not as complex as that of the maximum temperature. Overall, there is a continuous and obvious upward trend for minimum temperatures, and the upward trend in Shanghai is more obvious than that in Hong Kong.

### 3.6. Hot Days and Extremely Hot Days

**Figure 12:**
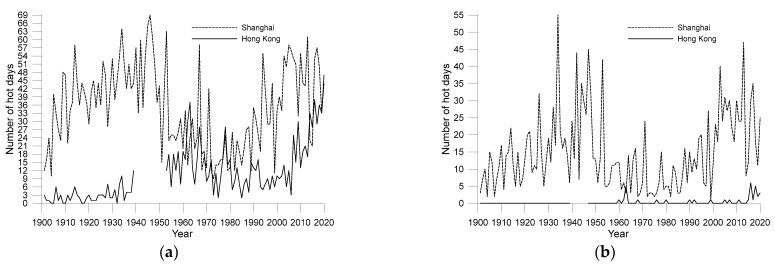
Total numbers of hot days (**a**) and extremely hot days (**b**) for the Shanghai and Hong Kong observatories.

Figure 12 shows the numbers of hot and extremely hot days for the two cities during the period from 1901–2020. A hot day is defined as a day with a daily maximum temperature higher than 33 °C, and an extremely hot day is defined as a day with a daily maximum temperature higher than 35 °C. Comparing Figure 12 with Figure 6, we can see that although the annual mean maximum temperature in Hong Kong is considerably higher than that of Shanghai, Shanghai has more hot days and extremely hot days than Hong Kong. This result is related to the geographical locations of the two cities; although they are both located in the subtropical monsoon climate zone, Shanghai is often covered by subtropical high pressure in midsummer, and therefore, it experiences more hot days and extremely hot days than Hong Kong. In addition, the location of SXO is farther away from coastal line than HKO, and the ocean plays a weaker role in regulating the temperature of SXO than that of HKO on a peninsula surrounded by ocean, which is another possible reason why Shanghai has recorded more hot days and extremely hot days. In most years in the last decade (except 2014), Shanghai recorded at least ten extremely hot days per year, whereas Hong Kong’s record was six extremely hot days, which occurred in 2016.

It is worth noting that small temperature changes had a dramatic effect on the number of hot and extremely hot days. Taking Shanghai as an example, the mean maximum temperature for the last ten years (2011–2020) was just 0.6 °C higher than that for the period from 1901–1930; however, the numbers of hot days and extremely hot days in the former period were greater by 4.9 and 8.9 days than the latter period, respectively. This finding shows that simply using the annual mean temperature rise rate to measure climate change is far from sufficient to explain the impact of urbanisation and global warming on local human settlements, especially in terms of climate comfort.

Another noteworthy feature is that the number of hot days, the number of extremely hot days, and the daily maximum temperature are closely related and show similar changes. For example, in Shanghai, the maximum temperature dropped between the late 1940s to the mid-1970s, and the number of hot days and extremely hot days decreased significantly during the same period. After 1980, as maximum temperatures began to rise, the number of hot days and extremely hot days increased rapidly, returning to the levels of the 1940s. The situation in Hong Kong was similar.

### 3.7. Differences between 1901–1930 and 1991–2020

Table 2 compares the average value of temperature-related elements in the last 30 years (1991–2020) with those for the period from 1901–1930. The differences in the current climate compared to more than a hundred years ago are noticeable. Table 1 shows that despite fluctuations over the past 120 years, the local climates of the two cities changed significantly. Both cities became relatively hotter, with increases in mean temperature, mean minimum temperature, and mean maximum temperature, while the DTR decreased.

However, the two cities exhibited certain differences. The increase in the maximum temperature of Shanghai was not as high as that of Hong Kong; however, increases in mean temperature and minimum temperature in Shanghai were higher than those in Hong Kong, and the DTR decline in Shanghai was far greater than that in Hong Kong. These differences are related to the latitudes of the two cities and the locations of their observation stations. Table 1 shows the negative impact of warming on the climate comfort of the two cities, as the number of hot days in Shanghai increased by 13.57%, while the number of extremely hot days increased by 76.07%. Although the increases in Hong Kong were more dramatic than those in Shanghai, they were mainly attributed to the low base of hot days and extremely hot days a century ago.

## 4. Conclusions

Using 120 years of surface observations, this study compared temperature trends of the two major East Asian cities of Shanghai and Hong Kong. These valuable long-term observations indicate local temperature changes in the two cities under the dual effects of global warming and urbanisation over more than a century. After statistical analysis and comparison of results, we drew the following conclusions:

(1) The average temperatures of the two cities underwent fluctuating increases during the past 120 years, with linear warming rates of 0.23 °C/decade in Shanghai and 0.13 °C/decade in Hong Kong. Both cities had an overall decrease in DTR, with a corresponding decrease in comfort in both cities during the subtropical heat of the summer months. The comparison of the average temperature of the two cities with the data of their adjacent rural regions shows that the impact of urbanization on the warming of Shanghai is greater than that of Hong Kong overall.
(2) The fluctuation ranges of maximum temperature in the two cities were considerably higher than those of mean temperature. In both cities, there was a phase of more than a decade when the annual mean maximum temperature decreased, i.e., there was no clear linear fit of annual mean maximum temperature. In contrast, the fluctuation ranges of minimum temperature were smaller, but the rates of linear increase were higher than those for mean air temperature, at 0.33 °C/decade in Shanghai and 0.15 °C/decade in Hong Kong.
(3) The DTR of the two cities decreased, and a certain phase of the decrease in DTR in the two cities was caused by a decrease in the maximum temperature rather than a rapid increase in minimum temperature. This study provides a conjectural explanation for the phasal decrease in the maximum temperature: at a certain stage of urban development, due to the shading effect of new high-rise buildings, the solar shortwave radiation reaching the Earth’s surface decreased, and the anthropogenic heat generated by the energy consumption of buildings and urban human activities at that time was not sufficient to make up for the reduced shortwave radiation; therefore, a phasal decline in the maximum temperatures of both cities occurred.
(4) High-quality observational environment metadata are important for understanding and interpreting long-term trends in urban air temperature. Long-term observational data in urban areas must be considered together with metadata analysis.
(5) The difference of the seasonal temperature variation is mainly determined by the difference of background climate induced by the difference of total solar radiation received at different latitudes. Additionally, the location of SXO is farther away from the coastal line than HKO, and the ocean plays a weaker role in regulating the temperature of SXO than that of HKO on a peninsula surrounded by ocean, which is another possible reason leading to the difference of the seasonal temperature variations.
(6) Analysis of trends for hot and extremely hot days shows that both cities experienced large changes in climate comfort over the past 120 years. Compared with a century ago, the numbers of hot days and extremely hot days in the two cities have changed significantly, which is not reflected in the discussion of the increase in mean temperature; therefore, they are a better indicator of the adverse impact of global warming on human settlements.

Finally, it should be noted that the quantitative evaluation results obtained in this study are based on the original raw data, while SXO had actually been relocated twice during the research period. In order to obtain a more accurate linear change trend, the homogenization of the original data needs to be further carried out, but this is beyond the scope of the discussion in this study.

## Figures and Tables

**Table 1:** Average relative error and correlation coefficient between observed and gridded data of Shanghai during the period of 1901 to 2020.

Data Groups **	Average Relative Error	Correlation Coefficient
A and B	1.90%	0.952442
A and C	2.02%	0.951391
A and D	1.99%	0.951637
C and D	0.11%	0.999997

** A: the original ground observation data of SXO; B: LSAT data of the grid cell where SXO is located; C: interpolated data from LSAT to position 1; D: interpolated data from LSAT to position 2.

**Table 2:** Comparison of the statistic of temperature-related data for the Shanghai and Hong Kong observatories.

Elements	Shanghai			Hong Kong		
	1901–1930	1991–2020	Change (%)	1901–1930	1991–2020	Change (%)
T_a_ (°C)	15.3	17.6	15.03	22.3	23.6	5.83
T_max_ (°C)	20.8	21.4	2.88	24.8	26.0	4.84
T_min_ (°C)	11.5	14.7	27.83	20.3	21.7	6.90
DTR (°C)	9.3	6.7	−27.96	4.5	4.3	−4.44
No. of hot days	36.1	41.0	13.57	2.1	20.2	861.90
No. of extremely hot days	11.7	20.6	76.07	0	1.0	∞

## Data Availability

The authors are not authorized to share the data.

## References

[1] Masson-Delmotte V.P., Zhai A., Pirani S.L., Connors C., Péan S., Berger N., Caud Y., Chen L., Goldfarb M.I., Gomis M. (2021). IPCC, 2021: Summary for Policymakers. Climate Change 2021: The Physical Science Basis. Contribution of Working Group I to the Sixth Assessment Report of the Intergovernmental Panel on Climate Change.

[2] Bai X., Chen J., Shi P. (2012). Landscape Urbanization and Economic Growth in China: Positive Feedbacks and Sustainability Dilemmas. Environ. Sci. Technol..

[3] Bai X., Shi P., Liu Y. (2014). Realizing China’s urban dream. Nature.

[4] Kalnay E., Cai M. (2003). Impact of urbanization and land-use change on climate. Nature.

[5] Masson V., Lion Y., Peter A., Pigeon G., Buyck J., Brun E. (2013). “Grand Paris”: Regional landscape change to adapt city to climate warming. Clim. Change.

[6] Grimmond S. (2007). Urbanization and global environmental change: Local effects of urban warming. Geogr. J..

[7] Li L., Chan P.W., Deng T., Yang H.-L., Luo H.-Y., Xia D., He Y.-Q. (2021). Review of advances in urban climate study in the Guangdong-Hong Kong-Macau Greater Bay Area, China. Atmos. Res..

[8] Oke T.R. (1981). Canyon geometry and the nocturnal urban heat island: Comparison of scale model and field observations. J. Climatol..

[9] Chen M., Liu W., Tao X. (2013). Evolution and assessment on China’s urbanization 1960–2010: Under-urbanization or over-urbanization?. Habitat Int..

[10] Yang X., Leung L.R., Zhao N., Zhao C., Qian Y., Hu K., Liu X., Chen B. (2017). Contribution of urbanization to the increase of extreme heat events in an urban agglomeration in east China. Geophys. Res. Lett..

[11] Sun Y., Zhang X., Ren G., Zwiers F.W., Hu T. (2016). Contribution of urbanization to warming in China. Nat. Clim. Change.

[12] Tse J.W.P., Yeung P.S., Fungb J.C.-H., Rene C., Wang R., Wong M.M.-F., CAI M. (2018). Investigation of the meteorological effects of urbanization in recent decades: A case study of major cities in Pearl River Delta. Urban Clim..

[13] Wu H., Gai Z., Guo Y., Li Y., Hao Y., Lu Z.-N. (2020). Does environmental pollution inhibit urbanization in China? A new perspective through residents’ medical and health costs. Environ. Res..

[14] Li L., Chan P.W., Wang D., Tan M. (2015). Rapid urbanization effect on local climate: Intercomparison of climate trends in Shenzhen and Hong Kong, 1968–2013. Clim. Res..

[15] Chen J., Li Q., Niu J., Sun L. (2011). Regional climate change and local urbanization effects on weather variables in Southeast China. Stoch. Environ. Res. Risk Assess..

[16] Chan H.S., Kok M.H., Lee T.C. (2012). Temperature trends in Hong Kong from a seasonal perspective. Clim. Res..

[17] Shun Djen C. (1992). The urban climate of Shanghai. Atmos. Environment. Part B. Urban Atmos..

[18] Zhang K., Wang R., Shen C., Da L. (2010). Temporal and spatial characteristics of the urban heat island during rapid urbanization in Shanghai, China. Environ. Monit. Assess..

[19] Cheng J., Li Q., Chao L., Maity S., Huang B., Jones P. (2020). Development of High Resolution and Homogenized Gridded Land Surface Air Temperature Data: A Case Study Over Pan-East Asia. Front. Environ. Sci..

[20] Lee T.-C. (2016). Metadata of Surface Meteorological Observations at the Hong Kong Observatory Headquarters 1884–2015.

[21] Hou Y.L., Chen B.-D., Yang X.-C., Liang P. (2013). Observed climate change in East China during 1961–2007. Adv. Clim. Change Res..

[22] Karl T.R., Kukla G., Razuvayev V.N., Changery M.J., Quayle R.G., Heim R.R., Easterling D.R., Fu C.B. (1991). Global warming: Evidence for asymmetric diurnal temperature change. Geophys. Res. Lett..

[23] Yang J., Liu H.-Z., Ou C.-Q., Lin G.-Z., Zhou Q., Shen G.-C., Chen P.-Y., Guo Y. (2013). Global climate change: Impact of diurnal temperature range on mortality in Guangzhou, China. Environ. Pollut..

